# PARP inhibitors in gastric cancer: unlocking precision oncology

**DOI:** 10.1093/oncolo/oyaf283

**Published:** 2025-09-17

**Authors:** Derek Tai, Vitor Goes, Sharanya Kumar, Pranati Shah, Farris Al-Manaseer, Daniel Park, Christiana Crook, Sofia Guzman, Xiaolin Zhu, Daneng Li, Dani Castillo

**Affiliations:** Department of Internal Medicine, Loma Linda University Medical Center, Loma Linda, CA 92354, United States; Department of Internal Medicine, Hospital Israelita Albert Einstein, São Paulo, SP 05652-900, Brazil; Department of Internal Medicine, Riverside University Health System, Moreno Valley, CA 92555, United States; Department of Internal Medicine, Loma Linda University Medical Center, Loma Linda, CA 92354, United States; Department of Internal Medicine, Loma Linda University Medical Center, Loma Linda, CA 92354, United States; Department of Internal Medicine, University of California San Francisco Fresno, Fresno, CA 93701, United States; Department of Medical Oncology and Therapeutics Research, City of Hope, Duarte, CA 91010, United States; Department of Medical Oncology and Therapeutics Research, City of Hope, Duarte, CA 91010, United States; Helen Diller Family Comprehensive Cancer Center, University of California San Francisco, San Francisco, CA 94115, United States; Department of Medical Oncology and Therapeutics Research, City of Hope, Duarte, CA 91010, United States; Department of Medical Oncology and Therapeutics Research, City of Hope, Duarte, CA 91010, United States

**Keywords:** PARP, gastric cancer, PARP inhibitor, homologous recombination deficiency, precision oncology

## Abstract

Gastric cancer (GC) demonstrates frequent alterations in homologous recombination repair (HRR) genes, and preclinical studies have demonstrated a clear synthetic lethality between HRR deficiency (HRD) and PARPi. While such preclinical synthetic lethality has translated into clinical benefits of PARPi in patients with HRD breast, ovarian, pancreatic, or prostate cancer, the therapeutic role of PARPi in GC remains unclear due to molecular heterogeneity and lack of validated biomarkers for patient selection. This review summarizes the mechanistic foundation for PARPi sensitivity in HRR-deficient GC tumors and evaluates emerging biomarkers, including genomic instability scores, *RAD51* foci formation, mutational signatures, and candidate genes such as *BRCA1/2*, *PALB2*, and *BARD1*. We highlight key clinical trials and ongoing research aimed at refining patient selection, optimizing combination strategies, and identifying predictive biomarkers. Improving biomarkers to identify *bona fide* HRD is essential to optimizing PARPi as a valuable treatment option for patients with GC. We outline a pathway for biomarker-guided adoption of PARPi in GC management. Early-phase clinical trials of PARPi monotherapy in GC have yielded limited efficacy, likely due to variable HRD status and other mechanisms of primary resistance. Combining PARPi with chemotherapy, immune checkpoint inhibitors, or anti-angiogenic agents offers strategies to potentially increase the tumor susceptibility to PARPi and overcome resistance.

Implications for Practice: Poly (ADP-ribose) polymerase inhibitors (PARPi) represent a potential treatment strategy in gastric cancer with homologous recombination repair deficiencies (HRD). Germline *BRCA1/2* mutations served as key biomarkers for PARPi responsiveness and regulatory approval in breast, ovarian, pancreatic, and prostate cancers, underscoring the importance of identifying *bona fide* HRD to maximize the clinical benefit of PARPi.

HRD tests rely mainly on genomic instability scores or HRR gene mutations due to a lack of validated functional assays. Further optimizing and tailoring these assays to detect *bona* fide HRD in GC holds promise to maximize the benefit/risk ratio of PARPi use in GC. Combination of PARPi with another anti-cancer agent may open more opportunities for fully exploiting the therapeutic potential of PARPi.

## Introduction

Gastric cancer (GC) is a leading cause of cancer-related mortality worldwide with limited treatment options and poor outcomes in advanced stages.^1^ Genomic profiling has identified homologous recombination repair (HRR) deficiencies in some GC tumors, including alterations in *BRCA1/2,* ataxia telangiectasia mutated (*ATM*), and *RAD51C*.^2–4^). These findings raise the potential for treating GC with poly (ADP-ribose) polymerase inhibitors (PARPi) which act through synthetic lethality, where simultaneous alteration of 2 genes leads to cell death, but alteration of either alone does not.^5–7^

While PARPi are approved for *BRCA*-mutated breast, ovarian, pancreatic, and prostate cancers,^8–11^ their clinical utility in GC remains investigational due to variability in homologous recombination deficiency (HRD) detection,^12–14^ inconsistent response rates,^15^^,^^16^ and resistance mechanisms.^17^^,^^18^ Biomarkers including genomic alterations, HRD scores, and functional assays are being evaluated for predicting response to PARPi, monitoring therapeutic response, and anticipating resistance.

This review explores the rationale for PARPi in GC, summarizes relevant clinical trials of monotherapy and combination approaches, and highlights emerging biomarkers. We also discuss challenges in patient selection and implementation of precision oncology in GC.

We searched PubMed and ClinicalTrials.gov for articles published between January 1, 2005, and June 30, 2025, using the terms “PARP inhibitors,” “gastric cancer,” “homologous recombination deficiency,” “resistance mechanisms,” and “precision oncology.” Peer-reviewed articles, clinical trial data, and conference abstracts were selected.

## Homologous recombination repair and PARP inhibitors

DNA damage occurs through single-strand breaks (SSB), double-strand breaks (DSB), and base modifications. HRR preserves genomic stability by repairing DSBs using a homologous DNA template. In healthy cells, HRR ensures high-fidelity DNA repair, whereas cancer cells often rely more on nonhomologous end joining, which is more error-prone and increases genomic instability.^19^^,^^20^

**Figure 1.**
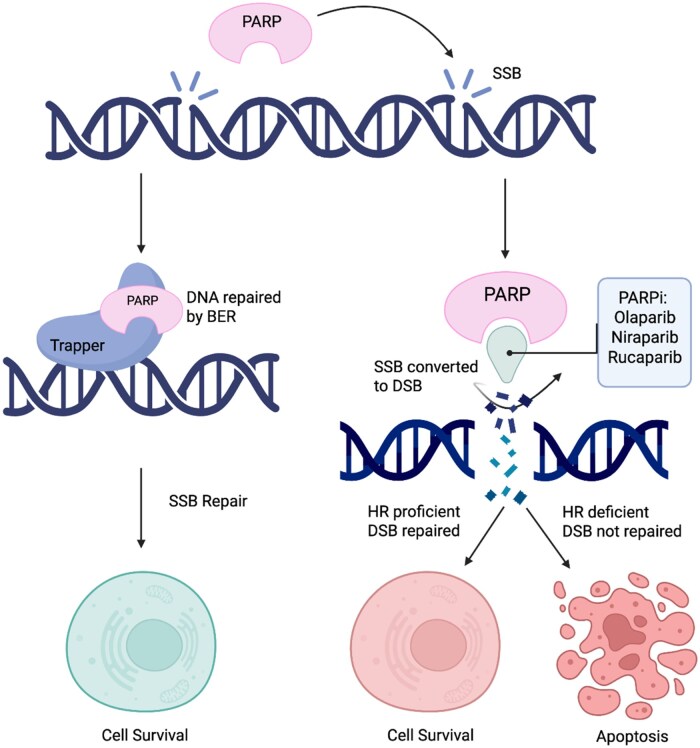
Mechanism of action of PARP inhibitors. Mechanism of action of PARP inhibitors (PARPi) in DNA repair and their selective cytotoxicity in HR-deficient cells. In HR-proficient cells, DSBs are repaired, leading to cell survival. In contrast, HR-deficient cells treated with PARPi accumulate DSBs, culminating in apoptosis. BER = base excision repair; DSB = double-strand break; HR = homologous recombination; PARPi = PARP inhibitor; SSB = single-strand break.

Poly (ADP-ribose) polymerases (PARPs) are enzymes involved in several cellular processes, including DNA repair.^21^  *PARP-1* is essential in SSB repair via base excision repair either by PARylation or by recruiting ADP-ribose polymers to attract repair proteins to the damage site. *PARP-1* also participates in HRR in HRR-proficient cells.^22^^,^^23^ When PARP is inhibited, SSBs accumulate and convert to DSBs during replication. In HRR-deficient cells, DSBs cannot be efficiently repaired, leading to cell death via synthetic lethality.^23^^,^^24^ PARPi can also cause *PARP*-*1* to become bound to sites of DNA damage, forming PARP-DNA complexes, a phenomenon known as PARP trapping.^24^  Figure 1 illustrates these mechanisms.

Germline mutations in *BRCA1/2*, the established genes involved in HRR, significantly increase the lifetime risk of developing breast, ovarian, pancreatic, and prostate cancers. Tumorigenesis in these patients often involves a second somatic hit that leads to *BRCA* inactivation and HRD. Preclinical models show that *BRCA1/2* mutations impair HRR, rendering tumor cells reliant on PARP-mediated repair, and thus, susceptible to PARP inhibition, a mechanism leveraged in the clinical use of PARPi in *BRCA*-mutated breast, ovarian, pancreatic, and prostate cancers.^5–7^^,^^20^^,^^25^

Multiple phase III trials have demonstrated the efficacy of PARPi in improving progression-free survival (PFS) and overall survival (OS), leading to FDA approval of PARPi in *BRCA*-mutated tumors. The SOLO1 trial demonstrated a median PFS of 49.9 months with maintenance olaparib in patients with newly diagnosed *BRCA*-mutated ovarian cancer vs 13.8 months with placebo.^26^ The OlympiAD trial in metastatic *BRCA*-mutated breast cancer reported a median PFS of 8 months on monotherapy olaparib vs 3.8 months on chemotherapy.^8^^,^^9^ The POLO trial reported a median PFS of 7.4 months on maintenance olaparib for patients with *BRCA*-mutated pancreatic cancer vs 3.8 months on placebo. Similarly, the PROfound trial showed improved radiographic PFS of 7.4 months with olaparib in metastatic *BRCA*-mutated prostate cancer vs 3.6 months with enzalutamide or abiraterone.^10^^,^^11^

## Homologous recombination deficiency in gastric cancer

Somatic mutations in HRR pathway genes such as *BRCA1/2 and RAD51C* occur in 10%-20% of GC.^2–4^ A case-control study of 10,705 Japanese patients with GC by Momozawa et al. revealed that germline pathogenic variants in *BRCA1/2* were associated with GC.^27^

Ichikawa et al. retrospectively evaluated 160 patients with metastatic GC treated with platinum-based chemotherapy in 2 Japanese institutions between 2009 and 2019. Targeted sequencing of primary tumor tissue revealed that 47 patients (29.4%) had alterations in at least 1 of 16 HRR-related genes. *RAD50* was the most frequently altered gene (7.5%), followed by *BRCA2* and *BRCA1* (6.3% and 5%, respectively). Patients with any HRR gene alteration had significantly better outcomes when treated with platinum-based therapy compared to patients with no HRR alterations (PFS 8.0 months vs 3.0 months, *P* = .010), raising the question whether these patients may also respond better to PARPi.^28^

Although not a conventional HRR gene, *ATM* plays a role in the broader DNA damage response. Preclinical data also link low *ATM* expression in GC cell lines to increased olaparib sensitivity, warranting further investigation into its role as a predictive biomarker.^29^

## Clinical testing for homologous recombination deficiency

Identifying HRD is important for predicting PARPi response, but assessing *bona fide* HRD remains challenging due to tumor heterogeneity, lack of standardization, and the lack of functional HRD testing in most clinically available assays. Next-Generation Sequencing (NGS) is commonly used to detect mutations and small indels and Single Nucleotide Polymorphism arrays detect copy number alterations and loss of heterogeneity, respectively.^12^^,^^30^ Myriad MyChoice CDx, the only FDA-approved HRD assay, assesses genomic instability, or genomic scars, using the Genomic Instability Score (GIS), which combines loss of heterozygosity, telomeric allelic imbalance, and large-scale transitions.^13^^,^^31^^,^^32^ While approved for ovarian cancer and under investigation in breast and prostate cancers, GIS reflects historical rather than real-time DNA repair capacity.^13^^,^^33^ However, tolerance to HRD may vary by tumor type, and HRD is ideally measured as a spectrum rather than a binary scale.^34–36^

To overcome the limitations of static GIS, functional assays have been developed to measure active DNA repair capacity. Functional assays such as *RAD51* nuclear foci measure active HRR, as *RAD51* is recruited to DSBs by the *BRCA1/*partner and localizer of BRCA2 (*PALB2*)/*BRCA2* complex.^37^ However, clinical use is limited by technical challenges, including immunofluorescence-based quantification.^38^ Nonetheless, the *RAD51* foci assay remains a stronger predictor of PARPi response with a positive predictive value of 93% vs 43%-50% seen in genomic HRD scores such as GIS.^39^

Current GIS and functional assays rely on tissue biopsies, which may not capture the full genomic landscape due to intrapatient heterogeneity. Moreover, genomic scars are static and may not detect dynamic changes such as reversion mutations that restore HRR and lead to PARPi resistance, highlighting the need for more dynamic and tumor-specific approaches to HRD testing.^14^ Liquid biopsies with the capacity to evaluate HRD, such as FoundationOne Liquid CDx, may help address this limitation,^14^ as it may capture circulating tumor DNA shed by multiple tumors. However, there are no FDA-approved assays for GC and limitations include higher risk of false-negatives (in the case of low tumor fraction) and potential false-positives (due to clonal hematopoiesis of indeterminate potential).^40–42^

## Emerging biomarkers in development for PARPi in gastric cancer

### Gene expression signature

Fujiya et al. developed a 100-gene expression signature predictive of PARPi sensitivity in 7 GC cell lines. This signature was partially informed by the presence of truncating mutations in HRR-related genes but was not a formal HRD score and was derived using supervised machine learning techniques comparing gene expression patterns between PARPi-sensitive and -resistant cell lines. In a cohort of 250 patients with gastroesophageal junction and gastric adenocarcinoma (GEA), 2.8% of patients showed a PARPi-sensitive profile. However, patients were not treated with PARPi and many genes in the signature were unrelated to HRR, suggesting the profile may reflect broader DNA damage response deficits rather than HRD specifically.^43^

### Mutational signature analysis and RAD51

In contrast, Prosz et al. used mutational signature analysis through The Cancer Genome Atlas and the Pan-Cancer Analysis of Whole Genomes and identified a higher prevalence (28%-40%) of HRD GEA tumors, even after excluding *BRCA-*mutated and MSI cases. This suggests traditional biomarkers may underestimate HRD prevalence in GEA. *RAD51* foci formation assays on signature-positive/negative GEA tumor samples confirmed that mutational signature expression correlated with defective HRR and increased PARPi sensitivity, underscoring the clinical potential of mutational signature profiling as an emerging biomarker to guide therapy in GEA.^44^ However, whole genome data is needed to derive this signature, precluding its clinical adoption.

These findings in GEA may cautiously extend to GC given their biological. Relying on single-gene biomarkers such as *BRCA1/2* or *RAD51* in GC has provided limited predictive power for response to PARPi, prompting increased interest in identifying a more accurate HRD phenotype through multi-gene expression signature, GIS, and mutational signatures, which may better predict therapeutic vulnerability to PARPi.^28^^,^^43^^,^^45^^,^^46^ Nonetheless, several genes, as highlighted below, remain biologically and clinically relevant and are under active investigation as potential markers of PARPi sensitivity.

### Ataxia telangiectasia mutated

*ATM* is a key regulator of DNA DSB repair.^29^ Although *ATM* expression loss has been proposed to sensitize tumors to PARPi, clinical validation remain inconsistent. A phase II study showed patients with low *ATM* protein expression treated with combination olaparib and paclitaxel experiencing a median OS of 13 months vs 8 months on paclitaxel alone.^47^ However, these findings were not confirmed in the subsequent phase III GOLD trial.^48^

Aside from the differential functional importance of *ATM* compared to the central HRR mediators like *BRCA1*/2, discrepancies may stem from limitations of the immunohistochemistry assay utilized to define *ATM* expression loss in GOLD, including intrapatient heterogeneity and antibody variability. Co-occurring mutations such as *TP53* mutations may also influence PARPi sensitivity; preclinical studies suggest combined *ATM* loss and *TP53* dysfunction can synergistically sensitize GC cells to PARPi.^49^

### Partner and localizer of BRCA2 and BRCA1-associated RING domain 1

*PALB2* and BRCA1-associated RING domain 1 (*BARD1*) are integral components of the HR pathway in DSB repair. *PALB2* bridges *BRCA1/2* to facilitate *RAD51* loading, while *BARD1* stabilizes *BRCA1* function. Loss-of-function mutations in either gene can cause HRD and increase reliance on PARP-mediated repair,^50^ though *PAL2B* mutations have demonstrated greater clinical relevance as a predictive biomarker.^51^^,^^52^ Regardless, both *PALB2* and *BARD1* mutations are included as eligibility biomarkers in ongoing PARPi trials in GC (NCT04692662, NCT04171700).

Additional genes potentially linked to PARPi sensitivity include *CHFR, CDK12, FANCA, CHEK1/2,* and *PTEN*.^53^ However, they are mechanistically distinct from HRD and their roles remain speculative, warranting further validation.

## PARPi monotherapy

Between 40% and 70% of patients with *BRCA1/2* mutations exhibit primary resistance or suboptimal response to PARPi monotherapy.^7^^,^^17^^,^^18^^,^^54^ In the phase IIIb LUCY trial, 252 patients with germline *BRCA*-mutated, *HER2*-negative metastatic breast cancer treated with olaparib monotherapy demonstrated a median PFS of 8.11 months and an objective response rate (ORR) of 48.6%, though the absence of a control arm limited direct efficacy assessment.^55^ In patients with *BRCA*-mutated ovarian cancer, responses to olaparib monotherapy were closely tied to platinum sensitivity. Patients with longer platinum-free intervals had higher response rates compared to patients who had disease progression on platinum-based therapy.^56^ Sandhu et al. reported a 40% response rate in 20 patients with *BRCA*-mutated metastatic castration-resistant prostate cancer who received niraparib monotherapy.^57^ Although these studies highlight meaningful responses, they also underscore that over half of biomarker-selected patients derive limited or variable benefit from PARPi monotherapy.

Khalid et al. conducted a phase II trial evaluating single-agent niraparib in 14 patients with metastatic HRD-positive GEA after progression on platinum-based therapy. No objective responses were observed and the study was terminated early due to poor accrual. Median OS and PFS were 6.6 months and 1.8 months, respectively, reflecting limited efficacy.^15^ Similarly, the phase II PARALLEL-303 trial assessed pamiparib maintenance therapy in 136 patients with advanced GC who responded to first-line platinum-based chemotherapy. Median PFS was 3.7 months with pamiparib vs 2.1 months with placebo; median OS was 10.2 months vs 12 months, suggesting limited clinical benefit of PARPi in unselected or molecularly heterogeneous patients with GC.^16^

Multiple mechanisms can cause PARPi resistance. One major cause is reduced reliance on HRR through reversion mutations in *BRCA1/2,* restoring the open reading frame and functional protein expression and reactivating HR, bypassing the synthetic lethality of PARPi.^54^ Other mechanisms include replication fork stabilization, loss of *PARP1* expression, and upregulation of drug efflux pumps such as *ABCB1*.^17^^,^^18^

Tumor heterogeneity increases treatment challenges as genetically diverse cell populations within an individual may display varying levels of sensitivity to PARPi and induce rapid emergence of resistance under treatment.^7^^,^^17^ These challenges emphasize the limited durability of PARPi monotherapy and highlight the need for combination strategies or next-generation inhibitors in precision oncology.

## PARPi combined with chemotherapy

Chemotherapy remains a cornerstone in advanced GC treatment. Preclinical studies in HRD or *BRCA-*mutated GC organoids demonstrated that combining PARPi and DNA-damaging agents may enhance cytotoxicity.^58^

A phase II trial comparing olaparib and paclitaxel vs paclitaxel alone in patients with metastatic GC showed no PFS benefit but improved OS with combination therapy vs placebo in both the overall population (median OS 13 vs 8 months) and an *ATM*-low subgroup (median OS not reached vs 8.2 months).^47^ However, the subsequent phase III GOLD trial,^48^ which randomized patients to olaparib and paclitaxel vs paclitaxel alone, showed a median OS of 8.8 months with combination therapy vs 6.9 months with paclitaxel alone (hazard ratio [HR] 0.79; 95% CI, 0.63-1.00; *P* = .026). This did not meet the pre-specified significance threshold (*P* < .025). Among patients with *ATM*-negative tumors, no significant OS benefit was observed (median OS 12.0 vs 10.0 months; HR 0.73; 95% CI, 0.43-1.25; *P* = .25). These results highlight the complexity of targeting DNA repair pathways in GC and suggest that *ATM* expression loss alone may not sufficiently predict PARPi benefit.

The study highlights the critical challenges in translating the synthetic lethality paradigm to GC. Unlike breast or ovarian cancers, where *BRCA* mutations serve as clear indicators of HRD, GC is molecularly diverse with alterations in multiple DNA repair pathways. This heterogeneity complicates patient selection and emphasizes the need for predictive biomarkers to determine PARPi therapy efficacy. PARPi effectiveness may be further hindered by issues related to drug penetration, tumor microenvironment, and compensatory DNA repair mechanisms.^18^

## PARPi combined with immune checkpoint inhibitors

DNA damage accumulated by PARPi can potentially increase tumor immunogenicity by increasing tumor-derived neoantigens and cytosolic DNA fragments, activating the *cGAS-STING* pathway and proinflammatory transcription factor NF-kB via *PARP1*.^59^^,^^60^ These mechanisms enhance the recruitment and activation of immune cells, potentially improving immune checkpoint inhibitors (ICI) efficacy.^61^

In preclinical models of *BRCA-*mutated breast and ovarian cancer, PARPi treatment upregulated expression of programmed death ligand 1 (PD-L1), creating an immunosuppressive microenvironment; ICI co-administration mitigated PARPi-induced immunosuppression.^32^^,^^61^ Moreover, defects in *BRCA1/2* and other HRD genes are known to increase T-cell infiltration and expression of *PD-L1* and *TMB* in multiple solid tumors and could serve as predictive biomarkers to identify GC patients most likely to have the greatest response to PARPi-ICI combination strategies.^28^^,^^62^^,^^63^

However, clinical data supporting this approach in GC are scarce. The phase II MEDIOLA basket trial evaluated patients treated with olaparib in combination with durvalumab with or without bevacizumab. Preliminary findings in the breast cancer cohort showed promising antitumor activity; data in the GC arm are pending.^64^ A phase I study by Xu et al. evaluated olaparib combined with an anti-*PD-L1* and anti-*LAG-3* bispecific antibody in patients with advanced GC, demonstrating good tolerability but modest efficacy.^65^ Further trials are needed to determine whether specific HRD-related immunologic profiles can predict good responses with PARPi-ICI combinations in GC.

## PARPi combined with anti-angiogenic agents

Anti-VEGF agents such as bevacizumab inhibit angiogenesis and induce intratumoral hypoxia, downregulating expression of HRR genes (including *BRCA1, BRCA2*, and *RAD51*) and inducing a transient HRD state.^66^^,^^67^ This hypoxia-induced HRD phenotype suggests that combining anti-angiogenic agents with PARPi could enhance synthetic lethality. Additional synergy may emerge from modifying molecular pathways such as *PI3K/AKT* signaling, further impairing DNA repair capacity and enhancing sensitivity to PARPi.^26^

The phase III PAOLA-1 trial enrolled 806 patients with advanced, high-grade ovarian cancer who responded to first-line platinum-based chemotherapy and were receiving maintenance bevacizumab; median PFS was improved in patients who received olaparib plus bevacizumab (46.8 months) vs placebo plus bevacizumab (17.6 months).^68^ This benefit was primarily driven by patients with HRD, regardless of *BRCA* mutation status. In contrast, patients with homologous recombination-proficient tumors had minimal to no benefit, reinforcing the requirement of HRD for PARPi sensitivity. Regardless, there may be some benefit in studying anti-VEGF agents with PARPi in the setting of GC given the success of PAOLA-1.^69^

A phase I study evaluated the combination of olaparib and the VEGFR2 inhibitor ramucirumab in patients with advanced GC. Ramucirumab was administered intravenously at 8 mg/kg every 2 weeks; olaparib was administered at the recommended phase 2 dose of 300 mg orally twice daily.^70^ Among 43 patients, the ORR was 14% (95% CI: 4.7-25.6) with a median PFS of 2.8 months (95% CI: 2.3-4.2) and median OS of 7.3 months (95% CI: 5.7-13.0). Although there was no formal comparator arm, numerical improvements in PFS and OS were observed in HRR-mutated tumors compared to non-HRR-mutated tumors. The combination was well-tolerated and showed greater efficacy than previously reported outcomes with single-agent ramucirumab, suggesting a potential therapeutic benefit in heavily pre-treated patients with advanced GC.^70^ Randomized trials are needed to confirm these findings.

## Emerging and next-generation PARPi

A new generation of agents is being developed to address current limitations of PARPi including resistance, limited efficacy, and off-target toxicity. These investigational compounds include *PARP1* inhibitors, dual-targeted agents, and molecules designed to modulate pathways such as *Wnt* signaling, angiogenesis, and epigenetic regulation. Table 1 summarizes these agents in preclinical and early clinical development.

**Table 1.** Novel and emerging PARP inhibitors.

Agent/compound	Targets	Development stage	Key mechanism/highlights
**Stenoparib (2X-121)**	PARP1/2, Tankyrase 1/2	Phase II	Tumor shrinkage and long-term stability in advanced ovarian cancer; favorable safety profile
**E7449**	PARP1/2, Tankyrase 1/2	Completed Phase I	Antitumor activity with durable disease control; inhibits Wnt/β-catenin and DNA repair simultaneously
**PPNR-4**	PARP1, Neuropilin-1 (NRP1)	Preclinical	Potent activity in breast cancer cell lines; minimal toxicity to normal cells
**III-16**	PARP1, BRD4	Preclinical	Novel dual-target agent; suppresses DNA repair and epigenetic readers; potent in pancreatic cancer models
**Compound 5a**	PARP1, EZH2	Preclinical	Epigenetic and DNA repair targeting; efficacy in triple-negative breast cancer models with wild-type BRCA
**Nesuparib**	PARP1/2, Tankyrase	Preclinical (GC model)	Active in gastric cancer models via HIPPO and Wnt pathway modulation

Stenoparib (2X-121) is a dual *PARP1/2* and *tankyrase1/2* inhibitor. A Phase II trial of heavily pre-treated patients with advanced ovarian cancer, including those with platinum-resistant and refractory disease, reported 5 out of 15 patients achieving stable disease for over 16 weeks, including one complete response. Notably, responses were observed in *BRCA-*wild-type tumors, suggesting activity beyond HRD through tankyrase inhibition within the *Wnt* pathway.^71^

E7449 inhibits *PARP1/2* and *tankyrase1/2*. In a phase I trial, E7449 demonstrated partial responses and prolonged stable disease in patients with advanced or metastatic tumors.^72^ Though early-phase and not biomarker-selected, the multi-targeted approach with dual inhibition of *PARP* and the *Wnt*/β-catenin pathway is particularly appealing for broader tumor types and heterogenous tumors like GC although this has yet to be validated in clinical settings.

PPNR-4 targets *PARP1* and *Neuropilin-1* (*NRP1*), a co-receptor implicated in tumor angiogenesis and immune evasion. PPNR-4 demonstrated potent antiproliferative effects in breast cancer cell lines while sparing normal tissues, offering a potential therapeutic index.^73^

III-16, a dual *PARP/BRD4* inhibitor targeting DNA repair and transcriptional regulation, showed preclinical efficacy in pancreatic cancer models, suggesting that co-inhibition of repair and transcriptional pathways may yield synergistic antitumor effects, particularly in therapy-resistant contexts.^74^

Compound 5a, a dual *PARP1/EZH2* inhibitor, suppresses tumor growth in triple-negative breast cancer models with wild-type *BRCA*, highlighting the potential to extend PARPi benefit beyond classical HRD subsets.^75^

Finally, nesuparib is a dual *PARP/tankyrase* inhibitor that has demonstrated activity in GC models by modulating both the *HIPPO* and *Wnt* signaling pathways, which are key regulators of cellular proliferation, differentiation, and apoptosis.^76^ These findings are particularly relevant in the context of GC, where *Wnt* pathway alterations and epigenetic dysregulation frequently contribute to tumor progression and treatment resistance.^76^

Collectively, these next-generation dual-target inhibitors represent an expanding frontier in precision oncology. Their mechanisms of action may enable efficacy in patients with HR-proficient or *BRCA* wild-type tumors, offering therapeutic options for patients who do not clinically respond to PARPi monotherapy. Further investigation is warranted to define their clinical role in GC.

## Ongoing clinical trials

Table 2 summarizes ongoing clinical trials evaluating PARPi.

**Table 2.** Ongoing trials evaluating PARPi in GC.

Trial ID/name	PARP inhibitor	Combination therapy	Phase	Endpoints	Key findings
**NCT02660034**	Pamiparib	Tislelizumab (anti-PD-1)	1a/1b	Safety, tolerability, pharmacokinetics, PFS	ORR: 20%; Median DoR: 17.1 months; Demonstrated acceptable safety and preliminary antitumor activity in advanced solid tumors including GC.
**NCT03150810**	Pamiparib	Temozolomide (TMZ)	1b	Safety, tolerability, MTD	MTD of TMZ: 60 mg/m² (7-day pulsed); Common TEAEs: anemia (56.1%), nausea (49.3%-54.5%), fatigue (47.9%-48.5%). Showed manageable safety profile and preliminary antitumor activity in advanced solid tumors.
**NCI100066**	Olaparib	Ramucirumab	1/2	Safety, efficacy, OS, PFS	Median PFS: 2.8 months; Median OS: 7.3 months; most common Grade ≥3 AEs: anemia, thrombocytopenia, neutropenia (54%). Combination was well-tolerated with efficacy exceeding historical controls in patients with heavily pre-treated metastatic GC.
**PARALLEL-303 (NCT03427814)**	Pamiparib	None (maintenance therapy)	2	PFS	Median PFS: 3.7 months (pamiparib) vs 2.1 months (placebo); Grade ≥3 TEAEs: 40.8% (pamiparib) vs 30.8% (placebo). Improved PFS compared to placebo in patients with inoperable locally advanced or metastatic GC.
**NCT04725994**	Venadaparib	Irinotecan	1b	Safety, efficacy	ORR 60%; PFS was not reached (1.2-21.5 months) in HRD patients
**NCT04209686**	Olaparib	Paclitaxel, Pembrolizumab	2	Safety, clinical activity	Study ongoing.
**NCT03026881**	Fluzoparib	Apatinib, Paclitaxel	1	Safety, tolerability	Study ongoing.
**NCT04511039**	Talazoparib	Trifluridine/Tipiracil	1b	Safety, MTD	Study ongoing.

Abbreviations: AE = adverse event; DoR = duration of response; GC = gastric cancer; GEJ = Gastroesophageal junction; MTD = maximum tolerated dose; ORR = overall response rate; OS = overall survival; PFS = progression-free survival; TEAE = treatment-emergent adverse event.

NCT02660034 is a Phase Ia/Ib trial evaluating the safety, tolerability, pharmacokinetics, and PFS of pamiparib combined with tislelizumab in patients with advanced solid tumors, including GC. Preliminary results include ORR of 20% and median duration of response of 17.1 months.^77^

NCT03150810 is a Phase Ib trial evaluating the safety, tolerability, and maximum tolerated dose (MTD) of pamiparib in combination with temozolomide in patients with advanced solid tumors, including GC. Preliminary findings identified the MTD of pamiparib as 60 mg once daily for 7 days followed by 14 days off per 21-day cycle in combination with standard-dose temozolomide. The combination showed manageable toxicity, supporting continued evaluation in selected solid tumor populations including GC.^78^

NCI10066 is a Phase I/II trial evaluating the safety and efficacy of combination olaparib plus ramucirumab in patients with metastatic GC. Preliminary results include median PFS of 2.8 months and median OS of 7.3 months.^70^

PARALLEL-303 is a Phase II trial evaluating pamiparib as a maintenance therapy in patients with inoperable locally advanced or metastatic GC. Preliminary results include a median PFS of 3.7 months on pamiparib compared to 2.1 months on placebo. OS data are immature.^16^

NCT04725994 is a Phase Ib trial evaluating safety and efficacy of venadaparib and irinotecan in patients with advanced GC, with an ORR of 60%; median PFS was not reached (95% CI: 1.2-21.5 months) in the subset of patients with HRD.^79^

Several ongoing early-phase trials are investigating PARPi-based combination strategies to overcome resistance and enhance efficacy in GC. NCT04209686 is a phase II trial evaluating a triple regimen of olaparib, paclitaxel, and pembrolizumab in previously treated advanced GC, targeting DNA repair, microtubule dynamics, and immune evasion in biomarker-unselected patients. NCT03026881 is a phase I trial assessing fluzoparib with apatinib and paclitaxel in patients with recurrent or metastatic GC. NCT04511039 is a phase I trial exploring talazoparib in combination with trifluridine/­tipiracil in patients with advanced gastroesophageal and colorectal cancers. Results from these studies are expected to inform future PARPi-based combination strategies across GC.

## Unanswered questions and limitations: patient-centered considerations

Despite promising early clinical data, several key questions remain before PARPi can be widely adopted in GC care. Which patients derive the greatest benefit from PARPi monotherapy or combination regimens? How can we better define HRD in GC, and are *BRCA1/2* mutations sufficient biomarkers for treatment selection? What is the optimal timing and sequencing of PARPi in relation to chemotherapy, immunotherapy, and anti-angiogenic agents? Current HRD assays are not validated in GC and vary widely in methodology, limiting their utility in routine practice. While *BRCA1/2* mutations are incorporated into many NGS panels, their predictive value outside of breast, ovarian, and prostate cancers remain uncertain. Furthermore, the majority of PARPi combination strategies lack robust prospective data to support their use in daily clinical practice.

Toxicity and quality-of-life outcomes are underreported in GC patients treated with PARPi. While these agents are generally well-tolerated in other solid tumors despite hematologic toxicities such as anemia, neutropenia, and thrombocytopenia, the long-term safety profile has not been systematically evaluated in GC. Most reported gastrointestinal side effects such as nausea and fatigue are low-grade and manageable. The impact of prolonged maintenance therapy on patient-reported outcomes, fatigue, and functional status remains unclear, emphasizing a continued need for prospective trials.

Finally, while PARPi remain investigational in GC, molecular testing for *BRCA1/2*, *RAD51* foci formation, *ATM, PALB2*, or broader HRR gene alterations may be considered for patients with advanced disease lacking other targetable options. Further evaluating the established and emerging biomarkers of HRD, specifically in GC, will improve patient selection to expand the therapeutic benefit of PARPi to a cancer type with an urgent unmet clinical need. Such biomarkers may also guide the development of combination therapies to maximize the potential of PARPi as a therapeutic option in GC.

## Conclusions

PARPi represent a promising therapeutic avenue in GC, particularly in tumors harboring *BRCA* mutations or broader HRD. However, current biomarkers lack precision and response rates remain heterogeneous. Despite these limitations, biomarker enrichment remains valuable in clinical trials to refine hypotheses, identify potential responders, and improve mechanistic understanding. Emerging combination strategies such as PARPi with immunotherapy, anti-angiogenics, or DNA-damaging agents offer potential and require validation in biomarker-enriched populations. As the molecular landscape of GC evolves, standardization of HRD testing and functional assays will be critical to refine patient selection. Future research must also address resistance mechanisms, long-term safety, and quality-of-life outcomes. Ultimately, integrating PARPi into GC care will depend on translating molecular insights into clinically actionable precision therapies.

## Data Availability

No new data were generated or analyzed in this study.
